# Reviews

**Published:** 1882-01

**Authors:** 


### Reviews.

**The Practice of Medicine and Surgery,** applied to the Diseases and Accidents incident to Women. By W. H. BYFORD, A. M., M. D , Professor of Gynecology in Rush Medical College, etc. Third edition, thoroughly revised and rewritten, with one hundred and sixty-four illustrations. Philadelphia: Lindsay & Blackiston. 1881.

The favor with which this valuable contribution to gynecology has been received by the profession, is best demonstrated by the fact that the third edition has been called for within a comparatively brief period. Prof. Byford's reputation as a writer in this specialty is the best guaranty that the work here offered is fully abreast with the more improved ideas and methods in the diseases and displacements of which it treats. This work, issued about the same time with Emmett's and Thomas', has passed the ordeal of comparative criticism, and is regarded as an equally valuable reference and reliable as an authority. This is the most flattering compliment that can be paid to the work, and to the profession which has utilized the opportunities offered by a western city and its surroundings, in the careful study and observation of an important department of medicine. We regard the library of the specialist incomplete which fails to contain this important contribution, and therefore recommend it to the profession.

---

**Indigestion, Biliousness and Gout in its Protean Aspects.**—Part I, Indigestion and Biliousness. By J. MILNER FOTHERGILL, M. D., Member of the Royal College of Physicians of London, etc., etc. New York: William Wood & Co. 1881.

The accomplished author presents in this work an exposition of functional disturbances of digestion and assimilation from a physiological standpoint. We have felt the want of such a work in the daily routine of practice, and find here a clear and logical discussion of disordered function, the most satisfactory of any yet published. Nothing could be expected of the writer after a perusal of previous contributions from his pen, but a valuable and thoroughly scientific production. It meets a want the practitioner often experiences, and will receive an appreciative response from the profession. We have not time to review its contents, but earnestly commend it as the best work on the subjects of which it treats, we have yet examined.

---

**The Wilderness Cure.** By MARC COOK, author of "Camp Lou." New York: William Wood & Co. 1881.

This little book of about one hundred and fifty pages, octavo, gives the experience of a consumptive patient in the Adirondack region. It includes a paper by Dr. Loomis, of New York, upon the advantages of this region to those affected with pulmonary disease, in which he gives the results of about fifteen cases. Of these but two fatal results are recorded, both tubercular phthisis. The great majority were decidedly benefited. Dr. Loomis speaks highly of this locality as a resort for those suffering from catarrhal or fibrous phthisis. The book is very interesting; this testimony is based on the fact that, on taking it up for the purpose of reviewing, we laid it down only when every word was read. The author's experience was remarkably satisfactory, and is detailed in a very entertaining manner. The book as a whole is very convincing as to the advantages of this resort.

---

**A Treatise on the Diseases of Infancy and Childhood.** By J. LEWIS SMITH, M. D., Clinical Professor of Diseases of Children in Bellevue Hospital Medical College, etc. Fifth edition, thoroughly revised, with illustrations. Philadelphia: Henry C. Lea's Son & Co. 1881.

That a book has reached the fifth edition is, per se, a sign, both that it has gaïned the favor and confidence of the profession, and that it deserves it. It is therefore hardly necessary to do more than to announce the appearance of the fifth edition. The author has rewritten portions of the book, and added especially to the therapeutics of the various diseases, but the work, although its usefulness is increased, is not materially enlarged by it.

---

**The Anatomist,** being a complete description of the anatomy of the human body, intended for the use of students preparing for examination at the Royal Colleges of Surgeons, and other medical bodies. Second edition improved and enlarged by the addition of 171 wood engravings. By M. W. HILLES, formerly lecturer on Anatomy and Physiology at the Westminster Hospital School of Medicine. New York: S. P. Putnam's Sons. 1881.

A convenient little compendium, by aid of which students may easily recapitulate the whole anatomy.

---

**Antiseptic Surgery: The Principles, Modes of Application and Results of the Lister Dressing.** By Dr. JUST LUCAS, Championniere Surgeon to the Hospital Tenon, etc. Translated from the second and completely revised edition, with the special sanction of the author, and edited by FREDERICK HENRY GERRISH, M. D., Surgeon to the Maine General Hospital, etc. Portland: Loring, Short & Hardon. 1881.

For more than a decade the antiseptic method of Joseph Lister has been known and appreciated on the continent. From England it has been introduced into almost every country, and uniformly the results have been the same, disappearance from the large hospitals of purulent infection as a complication of wounds, less frequency of erysipelas, rapid healing of wounds and a security and safety in operations, formerly considered dangerous or unjustifiable. In America the method is used only by the minority of surgeons, and its adversaries are many, often on account of disappointing results from incorrect use. We hail therefore with pleasure this little work, from which a perfect knowledge of the whole method may be acquired. A method by which such remarkable results as those of Volkman in Halle and many others have been accomplished, deserves to be known by every surgeon, and if we do not say, with Lorin, of Bale, "that every subject of amputation, who dies of purulent infection or erysipelas, is a victim of ignorance, lack of skill, or neglect of the surgeon," we shall still maintain, that only in the antiseptic method is there security, and that the surgeon, who does not use it, subjects his patients to an unnecessary and often fatal risk.

---

**A System of Surgery,** theoretical and practical, in treatises by various authors. Edited by T. HOLMES, M. A. Cantab, Surgeon and Lecturer on Surgery at St. George's Hospital, etc. First American, from second English edition. thoroughly revised and much enlarged. By JOHN H. PACKARD, M. D., Surgeon to Episcopal and St. Joseph's Hospital, Philadelphia. Assisted by a large corps of the most eminent American surgeons. In three volumes with many illustrations. Vol. II. Philadelphia: Henry C. Lea's Son & Co. 1881.

The second volume of Holmes's Surgery, which has just made its appearance, is divided into four parts. Part I, treats the surgical diseases of the organs of special sense; eye, ear, nose and tongue. Part II, the surgical diseases of the circulatory system; veins, arteries (including a long treatise of Holmes on aneurism) and the absorbent system. Part III contains treatises on diseases of the digestic tract, being diseases of the mouth, teeth, intestines, rectum and also hernia. Part IV gives descriptions of diseases of the genito-urinary organs, including diseases of urinary organs by Sir Henry Thompson, urinary calculi and lithotomy, lithotrity, diseases of the male organs of generation, gonorrhœa and surgical diseases of women. The articles are written by well known men, and revised by an able corps of American surgeons. The profession has therefore a guarantee that the work will represent the opinions of the best surgeons of both the old and new world.

---

**A Hand-Book of Uterine Therapeutics and Diseases of Women.** By EDWARD JOHN TILT, M. D. Fourth edition. New York: William Wood & Co., 27 Great Jones street. 1881.

The enterprising publishers of the Library of Standard Medical Authors could not have done a more valuable service to the American profession than in furnishing this valuable work on uterine therapeutics. It needs no introduction to our readers at our hands, while words of commendation are superfluous. Dr. Tilt is an acknowledged authority in the special field in which he has labored, and his views and opinions have always received careful consideration in all English-speaking nations. We regard this reprint a most valuable addition to the library of the practitioner, even if he makes no pretense to the special treatment of the class of diseases it discusses. The book is furnished in the usual style of this series, but in plainer print than some of the preceding volumes. It deserves the endorsement of the profession.

---

**The Science and Art of Midwifery.** By WILLIAM THOMPSON LUSK, A. M., M. D., Professor of Obstetrics, etc., Bellevue Hospital Medical College. With numerous illustrations. New York: D. Appleton & Co., 1, 3 & 5 Bond street. 1881.

The appearance of this work at this time will be regarded with special interest by the profession, for the reason that the high estimate formed of the late Prof. Elliot, wherever either the man or his writings were known, will naturally inspire a widespread interest in whatever proceeds from the pen of his successor in the obstretical chair of Bellevue. There is too marked a tendency to book-writing, and this is especially so among the profession in this country. Prof. Lusk's work, however, shows a careful and painstaking labor, with evidences that his rare opportunities have been industriously improved. The result, as unfolded in the work before us, is highly creditable to the author, who presents an accurate statement of the practice and art of midwifery, as existing at the present time, with all the modern improvements and investigations. We have been rather skeptical as to the necessity of further additions to the already numerous authorities upon midwifery, but, in carefully reviewing this work, there is found sufficient to warrant the author to enter upon its preparation, and we are gratified with the manner in which the task has been accomplished. The publishers present the work with fine paper and excellent type, making it especially attractive and worthy of a place in the library of the obstetrician. We are confident this fruit of the labor of Dr. Elliott's successor will maintain the high reputation which Bellevue has heretofore enjoyed, both as to the ability of its faculty and the professional soundness and erudition of their teachings,

---

**The Therapeutics of Gynecologv and Obstetrics,** comprising the Medical, Dietetic and Hygienic Treatment of Diseases of Women. Second edition, thoroughly revised and enlarged. Edited by WILLIAM B. ATKINSON, M. D., Lecturer on Diseases of Children at Jefferson Medical College, etc. Philadelphia: D. G. Brinton, 115 South Seventh street. 1881.

The great favor with which the medical profession received Naphey's Modern Therapeutics and Surgical Therapeutics, induced the enterprising publisher to undertake the compilation of a like work in the special department of gynecology and obstetrics, and Dr. Atkinson's wide experience was utilized in preparing this work, which rapidly passed through the first edition. The second edition is much improved by numerous corrections in the text, and also by the addition of nearly two hundred pages of new matter. The work is equally valuable with those to which it is really supplementary, although in an allied department of medicine, and furnishes to the practitioner a compendium which economizes his time, and supplies such data in regard to the special morbid and surgical conditions of which it treats, as are called for by the daily demands of an active professional life. A careful review of the work makes a most favorable impression of its merits, and enables us to commend it to the profession.

---

**A Manual of Ophthalmic Practice.** By HENRY S. SCHELL, M. D. Philadelphia: D. G. Brinton, Publishers.

The recent appearance of one or two other hand-books on the same subject seemed to make a new one entirely uncalled for. The writer of this, however, has chosen his facts with strict regard to their practical bearing, and arranged them in an attractive and instructive order, evidently intending to make it quite equal to any other work of similar size and scope. A very brief description of the anatomy of the eye introduces the reader to the subject, and then the various diseases are considered in detail. There is no striking originality in the manner of detailing the symptoms which accompany the different morbid conditions; and the methods of treatment advised, are usually such as have been confirmed by long experience. Moreover, the wood cuts, with which it is illustrated, are frequently recognized as figuring in older and larger books. But a manual is none the worse for presenting only classical views. Indeed, it should aim to present a clear and concise statement of the whole topic to those who are not already familiar with it, and as such, this one answers its purpose admirably. If a practitioner cares to study a case more thoroughly, he will turn to Wells, or Stellway, or other more complete treatises, but this little book is compact, it is practical, it is cheap, and thus recommends itself especially to students and to those busy in the details of professional routine.

---

**Eczema and its Management**—A Practical Treatise, based on the Study of two thousand five hundred cases of the Disease. By L. DUNCAN BULKLEY, A. M, M. D., Attending Physician for Skin and Venereal Diseases at the New York Hospital, etc. New York: G. P. Putnam & Sons, 27 and 29 West 23d street. London: J. & A. Churchill. 1881.

This important addition to the literature of dermatology represents the views and experience of the author, and in its pages there is observed, as a rule, an omission of references to the opinions and statements of other writers, the work consisting of personal experience, and the enunciations of principles, based upon wide observation, and careful and accurate study. The author takes the constitutional view of eczema, and many other diseases of the skin, a conversion from the views formerly held as to the local pathology of skin diseases, which he affirms is confirmed by daily experience and study. The work is a most valuable one, giving an exhaustive discussion of one of the most intractable of structural changes met with by the profession. To the practitioner, whose field of observation is limited, but who is called upon to combat a chronic eczema, the present work affords a resource, from which can be gleaned valuable experience and practical aid in therapeutics, while the pathology of eczema, herein enunciated, leads to a rational basis upon which to establish a successful treatment. We herald such works with pride, inasmuch as they demonstrate that American dermatologists are taking front rank in a most interesting and difficult department of medicine.

---

**Test-Book of Modern Midwifery.** By RODNEY GLISAN, M. D., Emeritus Professor of Obstetrics and Diseases of Women and Children in the Medical Department of the Williamette University, and late President of the Oregon State Medical Society. With one hundred and thirty illustrations. Price, cloth $4.00; sheep $5 00. Philadelphia: Presley Blakiston, 1012 Walnut Street. 1881.

The publication of a work in any department of medicine by a writer residing in the far west, especially on the Pacific coast, is reversing the order to which the profession has been heretofore accustomed, hence we have perused this work with quite a critical spirit, but with the impartiality which we endeavor to bring to this important journalistic duty.

In the preface the author pleads, as the necessity for the work he presents to the profession, the demand for "a work which shall more thoroughly represent American obstetric practice," and thus endeavors to convey the impression that the reprints of the works of Playfair and Leishman, and the translation of those of Cazeaux and Schroeder are too strictly European to be adapted to our western ideas. We fail to find where the excellent works to which we have referred, have been improved upon in the work under consideration. If it is characterized by an independence and vigor of expression, peculiar to the locality from which it originates, and by a boldness of practice and fertility of resource, the offspring of the free and vigorous spirit of the far west, we may expect to find a want of that fullness and accuracy, which the intellectual atmosphere of England and the Continent engenders. Prof. Glisan, however, presents a very excellent work. We doubt if it supplants the standard authors to which American obstetricians have been accustomed to refer. We commend it to the profession as an auxilliary to well-known authorities in this important department, not, however, fearing that its precepts will supplant the teachings of the old authors.

---

**Essentials of the Principles and Practice of Medicine.** By HENRY HARTSHORNE, A M , M. D. Fifth edition, thoroughly revised, and improved, with one hundred and forty-four illustrations. Philadelphia: Henry C. Lea's Sons & Co. 1881.

The former editions of the "Essentials of Practice" are probably better known and more widely distributed than any other medical work, with the exception, perhaps, of "Gray's Anatomy" and one or two similar works. This being the case, we have only to note the changes to be fonnd in the present edition. In the words of the author, " several hundred brief additions have been made throughout the work ; a number of new subjects have been written upon, especially in connection with the pathology of the nervous system; the illustrations have been considerably added to, and a large number of new and carefully selected formulæ have been introduced. An account is given, also, for the first time of the method of prescribing according to the metrical system; and a section is added upon eyesight, its examination and correction."

To bring within the space of six or seven hundred pages octavo an amount of information so large and of such widely extended scope, of course necessitates great brevity and condensation. It follows that upon no subject can this volume be expected to furnish an exhaustive treatise. To give the outlines of our present knowledge and beliefs regarding the broad subjects of disease and therapeutics is the evident object of the author. In this he is remarkably successful. To the general practitioner, who is fully abreast with the times, the book may not present much that is new. Still there are few such, and when we consider the daily and too rapidly increasing number of medical students, no one can doubt that the book finds a large field of usefulness.

---

**The Nurse and Mother:** a manual for the guidance of monthly nurses and mothers. By WALTER COLES, M. D., Consulting Physician to St. Ann's Lying-In Asylum. St. Louis: J. N. Chambers, Chicago, Ill., St. Louis, Mo., Atlanta, Ga. 1881.

The title gives a sufficient idea of the scope and objects of this little book. It is really a very valuable little work, giving all that is important for the mother and nurse to understand, and doing this in a clear, common sense manner, devoid of all the little superstitions which cling so tenaciously to the subjects herein treated. We strongly recommend it to the attention of physicians, who, it is hoped, will do the same to their patrons. Every physician appreciates the annoyance of being continually obliged to combat absurdities of belief and practice as exemplified in the majority of nurses and in even a large proportion of mothers. This book will, if generally circulated and read, tend greatly to dispel these vagaries.

---

**Library of Medical Classics.** New York: Benningham & Co., 1260 and 1262 Broadway.

We have received the first three numbers of these pamphlets, treating respectively upon diseases of the rectum, diseases of women and venereal diseases. The author of No. 1 is Henry Smith, T. R. C. S..; of No. 2, J. Mathews Duncan, M. D., LL.D., T. R. S. E., &c., and of No. 3, Berkeley Hill, Prof. of Clinical

Surgery in University College, London, &c., with the assistance of Arthur Cooper, late house surgeon to the Lock Hospital. The names of the several authors are a sufficient guarantee of the classical character of these monographs. They are issued in pamphlet form, the prices ranging from 20 to 30 cents. No. 3 has also been published by William Wood & Co., under the title of "The Students' Manual of Venereal Diseases." The practitioner is recommended to give these monographs his favorable consideration, as by so doing he can inexpensively obtain a library, very valuable and of a comprehensive character.

---

**Lectures on the Diagnosis and Treatment of Diseases of the Chest, Throat and Nasal Cavities.** By E T. Ingals, A. M., M. D., Lecturer on Diseases of the Chest and Physical Diagnosis, and on Laryngology in the Post Graduate Course, Rush Medical College, &c., &c. With one hundred and thirty-five illustrations. New York: William Wood & Co. 1881.

In the preface we find that "these lectures are designed to present a complete exposition of the subject of physical diagnosis so far as it relates to diseases of the chest, throat and nasal passages; to give the essential symptoms of each disease; to point out the symptoms and signs which are of most value in a differential diagnosis, and to outline briefly the proper treatment for the various affections. . . ." In the preparation of these lectures, the author says that he has availed himself of every source of information at his command, and he hopes that little has been overlooked which would be of value to the student and practitioner.

Our impression upon a more or less thorough examination of the book is that it fairly represents and elucidates the various subjects. The treatment of the sphygmograph is hardly equal to the importance of the subject. To those who have not already one of the many similar works, we recommend its purchase.

